# Current status of minimally invasive esophagectomy for esophageal cancer: Is it truly less invasive?

**DOI:** 10.1002/ags3.12224

**Published:** 2018-12-26

**Authors:** Taro Oshikiri, Gosuke Takiguchi, Susumu Miura, Nobuhisa Takase, Hiroshi Hasegawa, Masashi Yamamoto, Shingo Kanaji, Kimihiro Yamashita, Yoshiko Matsuda, Takeru Matsuda, Tetsu Nakamura, Satoshi Suzuki, Yoshihiro Kakeji

**Affiliations:** ^1^ Division of Gastrointestinal Surgery Department of Surgery Graduate School of Medicine Kobe University Kobe Japan; ^2^ Division of Minimally Invasive Surgery Department of Surgery Graduate School of Medicine Kobe University Kobe Japan; ^3^ Division of Community Medicine and Medical Network Department of Social Community Medicine and Health Science Graduate School of Medicine Kobe University Kobe Japan

**Keywords:** esophageal cancer, minimally invasive esophagectomy, thoracoscopic surgery, well‐experienced facilities

## Abstract

Esophagectomy with extended lymphadenectomy remains the mainstay of treatment for localized esophageal cancer. However, it is one of the most invasive procedures with high morbidity. To reduce invasiveness, minimally invasive esophagectomy (MIE), which includes thoracoscopic, laparoscopic, mediastinoscopic, and robotic surgery, is becoming popular worldwide. Thoracoscopic esophagectomy in the prone position is ergonomic for the surgeon and has better perioperative arterial oxygen pressure/fraction of inspired oxygen (P/F) ratio. Thoracoscopic esophagectomy in the left decubitus position is easy to introduce because it is similar to standard right posterolateral open esophagectomy (OE) in position. It has a relatively short operative time. Laparoscopic approach could potentially have a substantial effect on pneumonia prevention under the condition of thoracotomy. Mediastinoscopic surgery has the potential to reduce pulmonary complications because it can avoid a transthoracic procedure. In robotic surgery, in the future, less recurrent laryngeal nerve palsy will be expected as a result of polyarticular fine maneuvering without human tremors. In studies comparing MIE with OE, mediastinoscopic surgery and robotic surgery are usually not included; these studies show that MIE has a longer operative time and less blood loss than OE. MIE is particularly beneficial in reducing postoperative respiratory complications such as atelectasis, despite no dramatic decrease in pneumonia. Reoperation might occur more frequently with MIE. There is no significant difference in mortality rate between MIE and OE. It is important to recognize that the advantages of MIE, particularly “less invasiveness”, can be of benefit at facilities with experienced medical personnel.

## 1 INTRODUCTION

Torek reported the world's first case of transthoracic esophagectomy in 1913.^1^ It was carried out for a 67‐year‐old woman through the left thoracic cavity, with the proximal ends of the fourth through seventh ribs transected near their tubercles.^1^, ^2^ Food passed from a stoma in the proximal esophagus through an external tube to the gastrostomy.^1^ Since this first case, esophagectomy with extended lymphadenectomy remains the mainstay of treatment for localized esophageal cancer.^3^, ^4^, ^5^ However, it is one of the most invasive procedures and is associated with high morbidity.^6^, ^7^ One hundred years after the world's first case, minimally invasive esophagectomy (MIE) in the form of thoracoscopic and/or laparoscopic surgery is spreading around the world.^8^, ^9^, ^10^ Furthermore, mediastinoscopic and robotic surgery are being introduced as new MIE for esophageal cancer.^11^, ^12^ Some investigators have reported advantages of faster recovery and lower morbidity with MIE compared with open esophagectomy (OE).^13^ However, whether or not MIE is truly less invasive remains controversial. In the present review, MIE is defined as esophagectomy carried out using endoscopy, which provides a magnified view with decreased body wall destruction. MIE comprises several procedures, namely, thoracoscopic, laparoscopic, mediastinoscopic, and robotic esophagectomy. Moreover, “less invasive” is comprehended as fewer respiratory complications. The purpose of this review is to clarify the advantages and challenges of MIE.

## 2 ENDOSCOPIC SURGERY

### 2.1 Thoracoscopic surgery in the left decubitus or prone positions

#### 2.1.1 Genesis of thoracoscopic esophagectomy

Cuschieri et al^9^ first reported on thoracoscopic esophagectomy in the left decubitus position (TELD) in 1992. It has attracted attention as a potentially less invasive procedure. In the first case, an electronic pressure‐controlled carbon dioxide (CO_2_) insufflator was already being used with 8 mmHg of pressure to achieve lung collapse.^9^ As the positioning and approach for TELD and standard right posterolateral OE are almost identical, TELD became popular and spread worldwide quickly.^14^ Thoracoscopic esophagectomy in the prone position (TEP) was also reported first by Cuschieri et al in 1994.^10^ They carried out TEP for six patients in the full prone jackknife position. They described achieving excellent access to the mediastinum and the entire intrathoracic esophagus as well as good visual exposure because the right lung fell away from the operative field by gravity.^10^

#### 2.1.2 Learning curve for the thoracic procedure of MIE

There is a learning curve for both TELD and TEP. Osugi et al^15^ reported that there were significant differences between the first 36 cases and the 41 subsequent cases of TELD in terms of length of thoracic procedure, amount of blood loss, and pneumonia. Guo et al^16^ reported that at least 30 cases are needed to reach a plateau for TELD. After more than 60 TELD, lower morbidity could be achieved. Concerning TEP, Oshikiri et al reported on an individual surgeon's learning curve over the course of 100 procedures. They concluded that approximately 30‐60 cases are needed to reach a plateau for TEP and a lower morbidity rate.^17^ For both procedures, 30‐60 cases are needed to reach a plateau in the learning curve.

#### 2.1.3 Outcomes of TELD versus TEP

Many studies have compared TELD and TEP. Shen et al^18^ evaluated the surgeon's physical and mental stress during both procedures in a randomized control study. The drop in the eye‐blink rate of the surgeon at the end of the thoracic procedure from baseline was higher in the TELD group than in the TEP group (3.0 ± 1.4 blinks/min vs 1.2 ± 0.9 blinks/min, *P* < 0.001). The surgeon's symptom scale score was worse after TELD compared with TEP. The authors concluded that TEP is associated with a lighter workload and better ergonomic results than TELD.^18^ Noshiro et al^19^ also reported that TEP is associated with better surgeon ergonomics and better operative exposure than TELD because it is easier to explore the operative field around the left recurrent laryngeal nerve (RLN) during TEP. Mean duration of TEP was 307 minutes, which was significantly longer than the mean duration of TELD. However, the total estimated blood loss with TEP was significantly lower. There was no significant difference in the incidence of postoperative complications for the two procedures.^19^ Concerning postoperative oxygenation, some investigators reported that the TEP group had a significantly higher arterial oxygen pressure/fraction of inspired oxygen (P/F) ratio after surgery than the TELD group.^20^, ^21^ In contrast, no significant differences were observed in the frequency of respiratory complications.^20^, ^21^ In the TEP group, blood loss was significantly lower (*P* < 0.001), and the number of dissected intrathoracic lymph nodes was significantly higher (*P* = 0.03) than in the TELD group.^20^ In the TEP group, length of thoracic procedure was significantly longer and there was less blood loss.^21^ Comparison of short‐term outcomes between 54 cases of TEP and 33 cases of TELD showed that total and thoracic estimated blood loss, incidence of postoperative pulmonary complications, duration of intensive care unit (ICU) stay, and duration of hospital stay were significantly lower in the TEP group.^22^

Consequently, the advantages of TEP include better ergonomics, less blood loss, more dissected mediastinum lymph nodes, and a better P/F ratio. The advantages of TELD are similarity in positioning and approach as standard right posterolateral OE and shorter operative time than TEP. Concerning pneumonia, the advantage of TEP is controversial even though the perioperative P/F ratio is better with TEP. However, there were no significant differences between the TEP and TELD groups in the incidence of adverse events other than pneumonia.

### 2.2 Laparoscopic surgery

First, only laparoscopic surgery accompanied by the thoracic approach is discussed in this section. Laparoscopic transhiatal esophagectomy (THE) is discussed in the “Mediastinoscopic surgery” section.

In MIE, the effect of laparoscopic surgery in preventing pneumonia remains controversial. The thoracic procedure tends to be the main topic of discussion. However, the abdominal procedure should be just as important. The presence of open abdominal incisions can affect the rate of pulmonary complications and restrictive ventilatory impairment during the acute postoperative phase.^23^ Nozaki et al investigated the impact of laparoscopy on the prevention of pneumonia using data from Japan Clinical Oncology Group (JCOG) Study 0502. They concluded that laparoscopy failed to show any substantial effect on pneumonia prevention following thoracoscopic surgery.^24^ In contrast, Oshikiri et al^25^ reported that during the acute phase after TEP, hand‐assisted laparoscopic surgery (HALS) is associated with less restrictive ventilatory impairment and fewer subsequent pulmonary complications compared with open laparotomy. Glatz also reported the usefulness of laparoscopic surgery in esophagectomy.^26^ They compared hybrid minimally invasive laparoscopic‐thoracotomic esophagectomy (HMIE) with OE.^26^ Their analysis showed that HMIE is associated with a reduction in postoperative pulmonary morbidity, less perioperative blood loss, and shorter duration of hospital stay. This was essentially a comparison between laparoscopic and open laparotomy; the findings suggested that laparoscopic surgery might be advantageous.^26^

In conclusion, laparoscopic approach could potentially have a substantial effect on pneumonia prevention at least under the condition of thoracotomy.

### 2.3 Mediastinoscopic surgery

In this section, mediastinoscopic surgery indicates THE with mediastinoscope and/or laparoscope assistance.

As the conventional mediastinoscope has a specialized design for procedures involving a narrow operative field around the tip, it is unsuitable for radical esophagectomy with en bloc lymphadenectomy. In fact, use of a conventional mediastinoscope has been limited to esophageal mobilization with or without lymph node sampling in mediastinoscope‐assisted THE (MATHE).^27^, ^28^, ^29^ Fujiwara et al^30^ developed hand‐assisted laparoscopic THE with a systematic procedure for en bloc infracarinal lymph node dissection. In their procedure, transhiatal mobilization of the esophagus with lymph node dissection is done using a standardized method with hand‐assisted laparoscopic techniques. The cervical esophagus is mobilized using a left cervical approach. A total of 57 patients underwent esophagectomy, of whom 34 underwent the transthoracic procedure for upper mediastinal lymphadenectomy following esophagectomy and gastric tube reconstruction via the retrosternal route. Total operative time was significantly shorter in the laparoscopic THE group than in the laparoscopic THE with transthoracic procedure group (216 and 370 minutes, *P *<* *0.001). Blood loss in the laparoscopic THE group was less than that in the laparoscopic THE with transthoracic procedure group (139 and 238 mL, respectively), even though this difference was not statistically significant. However, fewer lymph nodes were retrieved in the laparoscopic THE group than in the laparoscopic THE with thoracic procedure group (24 and 39, *P *<* *0.001). The no residual tumor (R0) resection rate in both groups was similar. The incidence of RLN palsy was significantly higher in the laparoscopic THE with transthoracic procedure group, whereas there were no significant differences in the incidence of pneumonia between the two groups. The authors concluded that hand‐assisted laparoscopic THE, which includes a systematic mediastinal lymph node dissection, is safe and feasible as a type of MIE.^30^

Lymphadenectomy in the upper mediastinum is insufficient with laparoscopic THE alone, because the number of retrieved nodes was significantly lower than with laparoscopic THE followed by a thoracoscopic procedure.^30^ To overcome this disadvantage, Fujiwara et al developed a single‐port mediastinoscopic transcervical technique for upper mediastinal dissection.^31^, ^32^ A Lap‐Protector (FF07; Hakko, Tokyo, Japan) is inserted into the left cervical incision and an EZ Access port (Hakko) is attached. Mobilization of the esophagus with en bloc mediastinal dissection along the left RLN is then carried out with conventional flexible laparoscopy. After expansion of the intramediastinal space with CO_2_ insufflation, minute structures in the deep mediastinum around the aortic arch, bronchial arteries, nerves, and lymphatic vessels, are visualized clearly, allowing mediastinal dissection to be safely and carefully carried out along the nerve.^31^, ^32^ Tokairin et al^33^ also report that a similar technique was useful based on experience with Thiel‐embalmed human cadavers. They recommended carrying out both laparoscopic THE and transcervical mediastinoscopic lymphadenectomy under pneumomediastinum.^33^ In summary, Fujiwara et al developed MATHE by uniting transcervical mediastinoscopic lymphadenectomy to laparoscopic THE. They evaluated the safety of developed MATHE that consists of laparoscopic THE and single‐port transcervical mediastinoscopic lymphadenectomies in 60 patients.^11^ Median operative time and blood loss were 363 minutes and 235 mL, respectively. Two patients underwent conversion to thoracotomy. Postoperatively, pneumonia was observed in four patients (6.7%), although vocal cord palsy was more frequent (33%). Median number of resected thoracic lymph nodes was 21, and the R0 resection rate was 95%. They concluded that MATHE developed with the single‐port mediastinoscopic transcervical technique is feasible in terms of perioperative outcomes for a radical surgery to treat thoracic esophageal cancer, although its safety needs to be further investigated.^11^ Mori et al^34^ also carried out transcervical mediastinoscopic lymphadenectomy as part of totally non‐transthoracic radical esophagectomy for 17 patients in which the upper mediastinum and part of the middle mediastinum are dissected mainly with mediastinoscopic‐assisted surgery. There were no conversions to transthoracic procedure. Regarding short‐term outcomes, RLN palsy, chylous leak, and pulmonary complications were not observed. Median number of harvested lymph nodes from the upper mediastinal stations was 10. The authors concluded that transcervical mediastinoscopic lymphadenectomy is a safe and feasible procedure that enabled total non‐transthoracic radical esophagectomy in combination with a transhiatal approach.^34^

## 3 MINIMALLY INVASIVE ESOPHAGECTOMY VERSUS OPEN ESOPHAGECTOMY

In 2003, Luketich et al^14^ found that the mortality rate was lower (1.4%) and duration of hospital stay (7 days) was shorter for 222 patients who underwent TELD compared with most reports of OE. Biere et al^13^ conducted a multicenter, open‐label, randomized controlled trial at five sites in three countries to compare short‐term outcomes of TEP versus OE (Netherlands Trial Register, NTR TC 2452, TIME trial). The study found that TEP is associated with longer operative time, less blood loss, and a lower rate of pneumonia and RLN palsy than previously reported for OE.^13^ Long‐term oncological outcomes of the TIME trial were reported by Straatman et al.^35^ There were no differences in long‐term survival between the two groups. Combined overall 3‐year survival was 40.4% in the OE group versus 50.5% in the MIE group (*P* = 0.207). Three‐year DFS was 35.9% in the OE group versus 40.2% in the MIE group (Table 1). Based on the short‐term and long‐term results of the TIME trial, they concluded that MIE is useful for treating esophageal cancer.^35^ Yamashita et al^36^ compared short‐term and long‐term outcomes between 121 OE and 121 MIE using propensity score matching. All MIE consisted of TEP. Even though there was no significant difference in postoperative complications between the two groups, serum C‐reactive protein (CRP) levels during the first 3 and 5 postoperative days and peak CRP levels were significantly lower after MIE versus OE (MIE vs OE, median, 15.21 vs 19.50 mg/dL; *P* < 0.001). DFS and OS rates were significantly better in the MIE group than in the OE group (3‐year DFS rate, 81.7% vs 69.3%; log‐rank *P* = 0.021; 3‐year OS rate, 89.9% vs 79.2%; log‐rank *P* = 0.007) (Table 1). The authors concluded that MIE is an independent prognostic factor for patients with esophageal cancer.^36^ Takeuchi et al^37^ compared short‐term outcomes between MIE, including both TELD and TEP, and OE using a nationwide database in Japan. Results of operative time and blood loss were similar to the report by Biere et al.^13^ Concerning respiratory complications, the rate of atelectasis and the proportion of patients who required more than 48 hours of postoperative respiratory ventilation were significantly lower in the MIE group than in the OE group (3.6% vs 5.1%, *P *=* *0.002, and 8.9% vs 10.9%, *P *=* *0.006, respectively). However, there was no significant difference in the prevalence of pneumonia between the MIE and OE groups. In addition, the 30‐day reoperation rate was significantly higher in the MIE group than in the OE group (7.0% vs 5.3%, *P* = 0.004) (Table 1).^37^ Nozaki et al evaluated the safety profile of MIE, including both TELD and TEP, for T1bN0M0 esophageal cancer using data from JCOG Study 0502. They compared 109 OE and 101 MIE (Table 1).^38^ Seesing et al^39^ reported an analysis using propensity score matching of 433 OE and 433 MIE based on a nationwide registry in the Netherlands (Table 1). Consequently, a summary of Takeuchi, Nozaki, and Seesing's reports concluded that MIE is a safe procedure with similar mortality to OE and is particularly beneficial in reducing postoperative respiratory complications, but might be associated with higher reoperation rates (Table 1).^37^, ^38^, ^39^ Concerning long‐term health‐related quality of life, Barbour et al^40^ compared 110 OE with 377 MIE. Mean symptom scores for pain were significantly higher in the OE group than in the MIE group for 2 years after surgery (*P* = 0.036). In addition, mean constipation scores were significantly better for the MIE group at 3 months after surgery (*P* = 0.037). They concluded that OE is associated with more pain and constipation than MIE. Characteristics compared between OE and MIE are summarized (Table 2).

**Table 1:** Open esophagectomy vs minimally invasive esophagectomy

First author, year	Biere,^13^ 2012 Straatman,^35^ 2017 (TIME trial)	Yamashita,^36^ 2018	Takeuchi,^37^ 2017 (NCD in Japan)	Nozaki,^38^ 2015 (JCOG 0502)	Seesing,^39^ 2017
Study design	Multicenter open‐label randomized controlled trial	Propensity score matched analysis at a single institution	Propensity score matched analysis from a nationwide registry	Retrospective analysis from JCOG 0502	Propensity score matched analysis from a nationwide registry
Country	Netherlands	Japan	Japan	Japan	Netherlands
MIE position	Prone	Prone	Prone or left decubitus	Prone or left decubitus	Prone or left decubitus
No. of patients OE vs MIE	115 56 vs 59	242 121 vs 121	7030 3515 vs 3515	210 109 vs 101	866 433 vs 433
Operative time (min)	OE 299 min MIE 329 min *P *=* *0.002	OE 490 min MIE 615 min *P *<* *0.001	OE 461 min MIE 526 min *P *<* *0.001	OE 399 min MIE 510 min *P *<* *0.0001	NA
Blood loss (mL)	OE 475 mL MIE 200 mL *P *<* *0.001	OE 325 mL MIE 200 mL *P *<* *0.001	OE 608 mL MIE 442 mL *P *<* *0.001	OE 412 mL MIE 293 mL *P *<* *0.001	NA
Conversion	8 (14%)	NA	NA	NA	14 (3.4%)
Pneumonia	OE 16 (29%) MIE 5 (9%) *P *=* *0.005 *within 2 wks	OE 68 (56.2%) MIE 63 (52.1%) *P *= NS *All postoperative complications greater than C‐D Grade II	OE 553 (15.2%) MIE 490 (13.9%) *P *= NS	OE 17 (15.6%) MIE 8 (7.9%) *P *= NS	OE 148 (34.2%) MIE 154 (35.6%) *P *= NS *All pulmonary complications
Atelectasis	NA		OE 180 (5.1%) MIE 125 (3.6%) *P *=* *0.002	OE 24 (22.0%) MIE 11 (10.9%) *P *=* *0.041	
Anastomotic leak	OE 4 (7%) MIE 7 (12%) *P *= NS		OE 445 (12.7%) MIE 451 (12.8%) *P *= NS	OE 15 (13.8%) MIE 7 (6.9%) *P *= NS	OE 67 (15.5%) MIE 92 (21.2%) *P *=* *0.028
Recurrent laryngeal nerve palsy	OE 8 (14%) MIE 1 (2%) *P *=* *0.012		OE 285 (8.1%) MIE 361 (10.3%) *P *=* *0.002	OE 17 (15.6%) MIE 15 (14.9%) *P *= NS	OE 17 (3.9%) MIE 25 (5.8%) *P *= NS
Reoperation	OE 6 (11%) MIE 8 (14%) *P *= NS	NA	OE 188 (5.3%) MIE 247 (7.0%) *P *=* *0.004 *within 30 d	OE 2 (1.8%) MIE 10 (9.9%) *P *=* *0.016	OE 52 (12.3%) MIE 76 (18.0%) *P *=* *0.021 *Reintervention under general anesthesia
Duration of ICU stay (days)	OE 1 (0‐106) MIE 1 (0‐50) *P *= NS *median (range)	NA	OE 5.0 MIE 4.9 *P *= NS *mean	NA	OE 3 (0‐155) MIE 2 (0‐82) *P *= NS *median (range)
30‐d mortality	OE 0 (0%) MIE 1 (2%) *P *= NS	NA	OE 38 (1.1%) MIE 30 (0.9%) *P *= NS	OE 1 (0.9%) MIE 1 (1.0%) *P *= NS	OE 13 (3.0%) MIE 20 (4.7%) *P *= NS
Long‐term outcome	OE 40.4% MIE 50.5% *P *=* *0.207 *Three‐year OS rate	OE 79.2% MIE 89.9% *P *=* *0.007 *Three‐year OS rate	NA	NA	NA

C‐D, Clavien‐Dindo classification; JCOG, Japan Clinical Oncology Group; MIE, minimally invasive esophagectomy; NA, not applicable; NCD, National Clinical Database; NS, not significant; OE, open esophagectomy; OS, overall survival.

Asterisks mean annotations in each sections.

**Table 2:** Compared characteristics of open esophagectomy and minimally invasive esophagectomy

	OE		MIE
Operative time (min)	Shorter	<	Longer
Blood loss (mL)	More	>	Less
Respiratory complication	Equal or more	≧	Equal or less
Atelectasis	More	>	Less
Anastomotic leak	Equal or less	≦	Equal or more
Recurrent laryngeal nerve palsy	Equal	=	Equal
Reoperation	Less	<	More
Duration of ICU stay (days)	Longer	>	Shorter
30‐d mortality	Equal	=	Equal
Long‐term outcome	Equal	=	Equal

ICU, intensive care unit; MIE, minimally invasive esophagectomy; NS, not significant; OE, open esophagectomy.

Practically, some difficulties are encountered in the application of the satisfactory data from the high‐volume centers to low‐volume institutions. The reported data were results obtained at limited outstanding high‐volume facilities. Moreover, Nishigori et al^41^ reported that high‐volume hospitals had lower risk‐adjusted 30‐day and operative mortality rates compared with low‐volume hospitals. Data from the Japanese nationwide web‐based database included data on outcomes not only from high‐volume, but also from low‐volume hospitals, so that data from the Japanese nationwide web‐based database may not reflect the actual reduced invasiveness of MIE.^6^, ^37^ For example, lymphadenectomy around the RLN in MIE requires advanced skills to prevent nerve palsy, which may lead to pneumonia. In the Japanese nationwide web‐based database, RLN palsy rate was significantly higher for MIE than for OE.^37^ High RLN palsy rates in low‐volume centers may have an effect on the non‐significant decrease in the incidence of pneumonia in MIE. High reoperation rates in MIE were also similarly accounted for. Nozaki et al^38^ reported that mediastinal abscess, chylous leak from the thoracic duct, air leak from a bulla, and other complications causing reoperation were significantly greater in MIE. There is a possibility that on their learning curve for MIE, inexperienced endoscopic surgeons may have an increased reoperation rate.

In conclusion, most studies comparing OE and MIE had some limitations. Most were retrospective and limited to thoracoscopic surgery with or without laparoscopic surgery; mediastinoscopic surgery and robotic surgery were usually not evaluated. In particular, some studies included data not only from high‐volume but also from low‐volume centers. Considering these limitations, MIE is associated with longer operative time and less blood loss than OE. Concerning respiratory complications, MIE is particularly beneficial in reducing postoperative respiratory complications such as atelectasis, even though it is controversial as to whether MIE contributes dramatically to decreasing the incidence of pneumonia. For other complications such as anastomotic leak and RLN palsy, there is no significant difference between the two procedures. It is possible that reoperation or reintervention occurs more often with MIE. MIE is associated with less pain than OE. Regarding the mortality rate, there is no significant difference between MIE and OE. The dispersion of empirical value of each hospital for MIE may be an important reason for MIE not contributing markedly to decreasing the incidence of pneumonia and is, rather, associated with a higher reoperation rate.

## 4 ROBOTIC SURGERY

Horgan et al^42^ described robotic THE in 2003. In 2004, Kernstine et al^43^ reported their initial experience with the da Vinci Surgical System (DVSS; Intuitive Surgical, Inc., Sunnyvale, CA, USA) for thoracic esophagectomy with cervical, wide thoracic, and celiac axis lymphadenectomy and an esophagogastric anastomosis in the left side of the neck. In 2006, van Hillegersberg et al^44^ prospectively assessed 21 robot‐assisted MIE (RAMIE) using DVSS for mediastinal lymphadenectomy. In their series, 86% (18 patients) of esophagectomies were completed thoracoscopically. Operative time for the thoracoscopic procedure was 180 minutes, and median blood loss was 400 mL. Median number of retrieved lymph nodes was 20. Median durations of ICU stay and hospital stay were 4 and 18 days, respectively. Pulmonary complications rate was 48% (10 patients). Mortality rate was 5% (one patient) as a result of a tracheoesophageal fistula.^44^ Propensity score matching analysis of RAMIE versus conventional video‐assisted minimally invasive esophagectomy (VAMIE) was reported in 2018.^45^ Matching based on propensity scores produced 27 patients in each group. The RAMIE group had significantly longer operative time than the VAMIE group (349 and 294 minutes, respectively; *P* < 0.001), but similar blood loss volume (119 and 158 mL, respectively; *P* = 0.062). There was no significant difference between the two groups with respect to the mean number of dissected lymph nodes (20 and 19, respectively; *P *= 0.420), duration of postoperative hospital stay (13.8 and 12.7 days, respectively; *P* = 0.548), overall rate of complications (37.0% and 33.3%, respectively; *P* = 0.776), rate of RLN injury (14.8% and 11.1%, respectively; *P* = 1.000), and rate of pneumonia (18.5% and 7.4%, respectively; *P* = 0.224). The authors concluded that the short‐term outcomes of RAMIE and VAMIE are comparable, and that RAMIS is safe and feasible.^45^ van der Sluis et al^12^ reported the short‐term and long‐term outcomes in 108 cases of RAMIE. Pulmonary complications were the most common (36 patients, 33%). Median durations of ICU stay and postoperative hospital stay were 1 and 16 days, respectively. Mortality rate was 5%; 78% of patients presented with T3 or T4 disease, and 68% of patients had metastatic lymph nodes. Radical resection (ie, R0 resection) was carried out in 95% of patients. Median number of dissected lymph nodes was 26, median follow up was 58 months, 5‐year overall survival (OS) was 42%, median disease‐free survival (DFS) was 21 months, and median OS was 29 months. In 51 patients, tumor recurrences were confirmed. Locoregional, systemic, and combined recurrences were seen in six (6%), 31 (30%), and 14 (14%) patients, respectively. They concluded that RAMIE is effective oncologically, with a high percentage of R0 patients and adequate lymph node dissection. RAMIE showed good local control with a low local recurrence rate during long‐term follow up.^12^ Concerning the learning curve for RAMIE in a surgeon experienced in open and thoracolaparoscopic esophagectomy, Zhang et al^46^ found, using cumulative sum plots, that the learning curve for RAMIE would require operations on 26 patients and stomach mobilization would require operations on 14 patients. For the tableside assistant, experience of at least nine cases is needed to achieve an optimal technical level for thoracic docking and 16 cases for abdominal docking.^46^ A randomized controlled trial designed to compare RAMIE with OE as surgical treatment for resectable esophageal cancer (ROBOT trial) is ongoing (Dutch Trial Registry: NTR3291).^47^ The study started in January 2012. Follow up will be 5 years. Short‐term results will be analyzed and published after discharge of the last randomized patient.^47^

In conclusion, it is not clear whether RAMIE is better for reducing complication rates. A decreased incidence of RLN palsy leading to pneumonia will be expected in the future as a result of fine polyarticular maneuvering without human tremors. If RAMIE cannot reduce the incidence of RLN palsy and pneumonia, the significance of RAMIE will be controversial.

## 5 CONCLUSION

Minimally invasive esophagectomy is particularly beneficial in reducing the incidence of postoperative respiratory complications. Reoperation or reintervention might occur more often with MIE than with OE. The mortality rates for MIE and OE are similar. It is important to recognize that the advantages of MIE, particularly “less invasiveness”, can be availed at facilities with experienced medical personnel.

## CONFLICTS OF INTEREST

Authors declare no conflicts of interest for this article.
